# Editorial: Dimensional assessment of personality disorders in young people: A closer look on personality functioning in younger ages, different cultures, and various clinical settings

**DOI:** 10.3389/fpsyt.2022.1082189

**Published:** 2022-11-22

**Authors:** Klaus Schmeck, Hojka Gregoric Kumperscak, Marc Birkhölzer, Kirstin Goth

**Affiliations:** ^1^Department of Clinical Research, Medical Faculty, University of Basel, Basel, Switzerland; ^2^University Hospital Maribor, Maribor, Slovenia; ^3^Department of Child and Adolescent Psychiatric Research, Psychiatric University Hospitals Basel, Basel, Switzerland

**Keywords:** personality functioning, dimensional assessment, maladaptive traits, personality disorder, identity, adolescence

## Introduction

From a developmental perspective, adolescence is a critical period to intervene in order to alter developmental trajectories (1). This is especially true for mental problems, as adolescence is the period in life where psychiatric disorders contribute the most to morbidity in comparison to somatic diseases. Personality disorders (PD) in general and Borderline personality disorder (BPD) in particular are among the most severe mental health problems, as they are associated with poor psychosocial functioning, poor physical health, increased psychiatric comorbidity, and enormous societal costs (2). The Global Alliance for Prevention and Early Intervention for Borderline Personality Disorder (2) was founded to promote early detection and early intervention for BPD in young people as there is still reluctance to use this diagnosis in adolescents even if there is sufficient knowledge that BPD can be diagnosed in young people reliably and validly (2, 3). The next decade will reveal if the major changes in ICD-11 with the application of a life-span perspective on all mental disorders will lead to a substantial change in classification routines. Currently, as a consequence of not being diagnosed correctly, adolescents suffering from BPD are deprived of effective empirically based treatments (4), which increases the risk of an impaired life-course, since we know that untreated BPD symptoms in late childhood are predictive of a poor outcome in all areas of life (5) which increases the risk of chronic disability.

Early detection requires assessment tools with appropriate psychometric properties that are designed to capture the core elements of personality pathology according to the new classification systems. The alternative model of Personality Disorders (AMPD) in the DSM-5 (6) introduced a dimensional approach to assess an overall measure of PD severity (Criterion A). Four dimensions of personality functioning are supposed to describe the core impairments of PD: Identity, Self-Direction, Empathy, and Intimacy. In ICD-11 (7), the basic definition of PD is mostly similar to the DSM-5 AMPD, with impairments of self- and interpersonal functioning as core criteria of PD. The clinician is also allowed to assign one or more trait domain specifiers that contribute to the individual expression of personality disturbances (i.e., *Negative Affectivity, Detachment, Dissociality, Disinhibition, Anankastia*). Finally, with the aim of facilitating the identification of individuals who may respond to established treatments, a *Borderline Pattern specifier* has been included, which is essentially based on the DSM-5 Borderline PD diagnostic criteria.

The Operationalised Psychodynamic Diagnosis (8) provides a similar model to assess the severity of a patients' structural impairment, using four dimensions of personality structure: Control, Identity, Interpersonality, and Attachment.

In order to investigate the potential of these dimensional models to detect emerging personality disorders in children, adolescents and young adults, and to promote early detection and early intervention (in line with the GAP agenda), we established an international working group of child and adolescent psychiatrists and psychologists who are interested to develop reliable and valid assessment tools specifically adapted for younger ages and various cultural contexts.

The present Research Topic “Dimensional Assessment of Personality Disorders in Young People: A Closer Look on Personality Functioning in Younger Ages, Different cultures, and Various Clinical Settings” is a collection of articles that represent the work of research groups who promote the early detection of personality pathology in young people aged 12 to 26. The eleven articles of authors from eight different nations cover a broad range of aspects that are essential for the early assessment of personality functioning and pathology.

## Conceptual aspects of BPD in adolescence

Disturbed identity development has been identified as an important feature of maladaptive personality functioning as represented in DSM-5 and ICD-11. In their contribution, Sharp et al. demonstrated the course of maladaptive personality functioning in the domain identity during adolescence in a large community sample of 2,381 adolescents. Results of this study suggest a normative increase in maladaptive identity development after age 12 (although not touching clinically relevant levels), which remained consistent until age 17 when it dropped back to levels observed in 12-year-olds. Important for the understanding of the development of impaired personality functioning (9) is the result that maladaptive identity development was significantly associated with mean-level increases in borderline personality features, and that, with increasing age, these constructs become more closely associated.

The significance of impaired identity in Borderline personality disorder is also in the focus of the work of Rivnyák et al.. In a sample of 169 adolescents from the general population they used network analysis to test the importance of identity diffusion in the organization of borderline personality features. The main result was that in this network the shortest paths from one specific borderline feature to another specific borderline feature went through identity diffusion. This result emphasizes the central role of identity diffusion as a core symptom of Borderline Personality disorder beyond affect dysregulation.

Barkauskiene et al. broadened the perspective by focussing on the full scope of personality functioning (criterion A) and also on maladaptive personality traits (criterion B). In a mixed sample of 568 adolescents from the community, clinical settings and youth forensic care, the authors explored the associations of Criterion A (assessed with the LoPF-Q 12–18 questionnaire Lithuanian version) and B (assessed with the PID-5-BF) and their contribution in predicting borderline personality features in young people. The strongly interrelated criterion A and B were both significant predictors of borderline personality features in adolescents. However, there was an incremental value of criterion A (all four domains of personality functioning identity, self-direction, empathy, intimacy) over both criterion B and general psychopathology to capture the core features of borderline personality in young people.

### Assessment of identity and personality functioning in adolescence

If impaired identity and personality functioning play such a crucial role in the understanding of (adolescent) Borderline pathology, the reliable and valid assessment of those constructs is of high clinical relevance. Four papers of the present Research Topic focus on aspects of reliability and validity in the assessment of identity and personality functioning.

Rivnyák et al. evaluated the factor structure, complex relation, and validity of two measures assessing identity processes and identity statuses in a Hungarian adolescent sample: the Dimensions for Identity Development Scale (DIDS) and the Utrecht-Management of Identity Commitments Scale (U-MICS). Results particularly support the use of the construct Commitment which is part of both inventories, showing negative correlations with internalizing and externalizing problems and positive correlations with adaptive cognitive emotion regulation strategies and self-esteem.

Sarrar and Goth investigated the assessment of personality functioning from a psychodynamic perspective. They introduced an age-adapted version DSQ-22-A (Defense Style Questionnaire) for adolescent self-report and investigated its relation to the structure questionnaire of the Operationalized Psychodynamic Diagnosis in childhood and adolescence [OPD-CA2-SQ; (10)] and to aspects of psychopathology in a combined clinical and school sample of 396 adolescents. Results suggested the particular validity of the scale Maladaptive Defenses, consisting of the defense mechanisms autistic fantasy, affect isolation, projection, somatization, and splitting. However, fundamental changes concerning some basic operationalizations of the defense mechanisms and the 2-item-method were suggested for international discussion.

Two papers focus on the psychometric properties of the questionnaire AIDA [Assessment of Identity Development in Adolescence; (11, 12)], that, up to now, has been translated to 26 languages worldwide.

González Flores et al. adapted the AIDA for a Panamanian population. The AIDA Panama showed excellent internal consistency, the total scale Identity Diffusion showed high covariations with psychopathology (SDQ) and immature defenses (DSQ). Bifactorial CFA support the existence of a general factor and the unidimensionality of the questionnaire. This corresponds to Sharp et al. who demonstrated that the AIDA items appear to be best represented by a single latent factor with a good fit in a CFA.

Plakolm Erlač et al. used sophisticated methods to study both the implicit and explicit self-concept of identity diffusion in a sample of adolescent patients with BPD by using an implicit association task (IAT) and the Slovenian version of AIDA. Self-report based AIDA scores to denote impaired identity functioning were significantly correlated with the implicit measure of identity diffusion. However, when looking at the predictive ability of implicit and explicit measures, only explicit identity diffusion (according to the AIDA scores) was significantly associated with borderline features.

### Impairment in personality functioning in different populations

Currently, the impact of gender identity on psychological wellbeing is one of the most controversial issues in many scientific areas including child and adolescent psychiatry. In ICD-11, gender dysphoria was taken out of the spectrum of psychiatric diagnoses to demonstrate that struggling with gender orientation is not equivalent to having mental problems. In clinical settings however, it is of major importance to differentiate gender dysphoric adolescents with no signs of mental illness from those individuals with comorbid gender dysphoria and personality disorders. In a Finnish sample using the AIDA questionnaire, Karvonen et al. compared the identity integration of 215 adolescents with features of gender dysphoria, 400 adolescents from general population and 77 adolescent psychiatric outpatients. Results of the study were clear in the direction of higher levels of identity diffusion in adolescent psychiatric outpatients compared to adolescents with features of gender dysphoria whose scores were similar to adolescents from the general population.

In a German study, Zettl et al. focused on a largely understudied group by examining identity development and maladaptive personality traits in 120 young adult refugees from 22 countries compared to 281 adults with first- or second-generation migration background. The personality functioning domain identity was assessed with a short version of AIDA in culturally adapted versions (English, Persian, Arabic, Turkish, Croatian, French, and German), and the Personality Inventory for DSM-5 – Brief Form (PID-5-BF) was used to assess criterion B. Compared to migrants, refugees reported significantly higher levels of identity diffusion, negative affectivity, detachment, antagonism, and disinhibition, demonstrating the burden of displacement on personality development.

### Trajectories of personality functioning across the life-span

Finally, two articles of the Research Topic are focused on personality and impaired personality functioning across the life-span. As part of the Preschool Child Development Trajectory Study, Paulus et al. examined the predictive value of temperament measured in preschool age (mean age 4.2 years) for psychopathology later in childhood (mean age 9.2 years). Preschool temperament contributed differently to the development of externalizing and internalizing problems in middle childhood. High levels of frustration and anger in the preschool age were strong predictors of impaired mental health at age nine.

In a longitudinal design, d'Huart et al. studied both prevalence and 10-year stability of personality disorders from adolescence (mean age 15.8 years) to young adulthood (mean age 25.9 years) in a high-risk sample of 115 individuals with a history of residential child welfare and juvenile-justice placements in Switzerland. Prevalence of personality disorders was 20.0% at baseline and 30.4% at follow-up. The mean-level stability of any personality disorder was only moderate, and the mean-level stability of specific personality disorders was even low. These results support the overwhelming evidence of numerous studies that the stability of personality disorder diagnoses, which has been seen as the core of the disorder for a long time, is far lower than expected. This insight has penetrated the concept of personality disorders in the upcoming ICD-11.

### Future direction

We hope that the eleven articles of this Research Topic shed light upon the relevance of assessing impaired personality functioning in general and identity diffusion more specifically. As Sharp (9) has outlined, criterion A, i.e., impairment in personality functioning, seems to be the core of personality dysfuntion in adolescence that can lead to long-lasting disability if not treated properly. With our work, we want to foster the aims of the Global Alliance for Prevention and Early Intervention for Borderline Personality Disorder (GAP) in the fight for a better support of young people with early emerging personality disorders, and we strongly agree with GAP that this should be a major public health priority. With our newly developed test versions LoPF-Q Parent 6–18, LoPF-Q Therapist 6–18, OPD-CA2-SQ Parent 6–18 and PID5BF+ CA IRF we will start to investigate the possibility of an even earlier detection of (beginning) personality difficulties and disorders in parent report for children from 6 years up in a longitudinal setting. From 2023 on, our EARLY study is starting with project partners from 12 countries.

## Author contributions

KS wrote the first draft of the manuscript. All authors contributed to manuscript revision, read, and approved the final version.

## Conflict of interest

Authors KG, MB, and KS are of assessment instruments that are used in the research of some of the articles in this collection (KG and KS: AIDA and OPD-CA2-SQ; KG, MB, and KS: LoPF-Q 12-18). The remaining author declares that the research was conducted in the absence of any commercial or financial relationships that could be construed as a potential conflict of interest.

## Publisher's note

All claims expressed in this article are solely those of the authors and do not necessarily represent those of their affiliated organizations, or those of the publisher, the editors and the reviewers. Any product that may be evaluated in this article, or claim that may be made by its manufacturer, is not guaranteed or endorsed by the publisher.
